# The coagulation–inflammation axis in advanced cancer: associations with cardiovascular-thrombotic complications

**DOI:** 10.1007/s11239-026-03241-3

**Published:** 2026-02-02

**Authors:** Charlotte Wagner, Fiona Bruns, Claudia Maletzki, Annabell Wolff, Moosheer Alammar, Anett Seifert, Ursula Kriesen, Karen Rischmüller, Sonja Oehmcke-Hecht, Christian Junghanss

**Affiliations:** 1 Department of Internal Medicine, Clinic and Polyclinic for Hematology, Hemostaseology, Oncology, Stem Cell Therapy, and Palliative Medicine, Rostock University Medical Center, University of Rostock, Ernst-Heydemann-Str. 6, D-18057 Rostock, Germany; 2 Department of Medical Interdisciplinary Studies, Institute of Medical Microbiology, Virology and Hygiene, Rostock University Medical Center, University of Rostock, 18057 Rostock, Germany; 3 Department of Internal Medicine, Clinic and Polyclinic for Gastroenterology, Hepatology, and Nutritional Medicine, Rostock University Medical Center, University of Rostock, 18057 Rostock, Germany

**Keywords:** Palliative cancer, Cardiovascular-thrombotic complications, Coagulation dysregulation, Extracellular vesicles, Anticoagulation

## Abstract

Cardiovascular–thrombotic complications (CVTCs) are common in advanced malignancies and contribute substantially to morbidity and mortality. The relationships between inflammation, coagulation abnormalities, and extracellular vesicle (EV) profiles in palliative-stage disease remain insufficiently understood. In this exploratory cross-sectional study, clinical characteristics, coagulation parameters, inflammatory markers, and EV profiles were assessed at a single time point in 80 patients with advanced solid tumors receiving palliative care. Patients with a history of CVTCs were compared with those without. Patients with CVTCs showed higher levels of IL-6 and CRP, prolonged coagulation times, and lower activities of several coagulation factors, including FII, FVII, FX, FXII, and FXIII. Fibrinogen and d-dimer levels were elevated in the overall cohort and were higher in patients with CVTCs. Exploratory regression analyses indicated associations between lower FII activity, higher platelet counts, older age, and the presence of CVTCs. Cancer patients exhibited higher concentrations of small EV–like particles and shorter EV-associated clotting times compared with healthy donors; however, EV-related measures did not differ between patients with and without CVTCs. Palliative cancer patients demonstrate heterogeneous alterations in inflammatory and coagulation parameters, with more pronounced abnormalities in those with CVTCs. EV concentrations were elevated overall but did not distinguish between clinical subgroups. These findings are descriptive and hypothesis-generating, underscoring the need for longitudinal studies to clarify temporal relationships, potential mechanistic pathways, and the clinical relevance of coagulation and EV-associated alterations in advanced cancer.

**Graphical Abstract:**

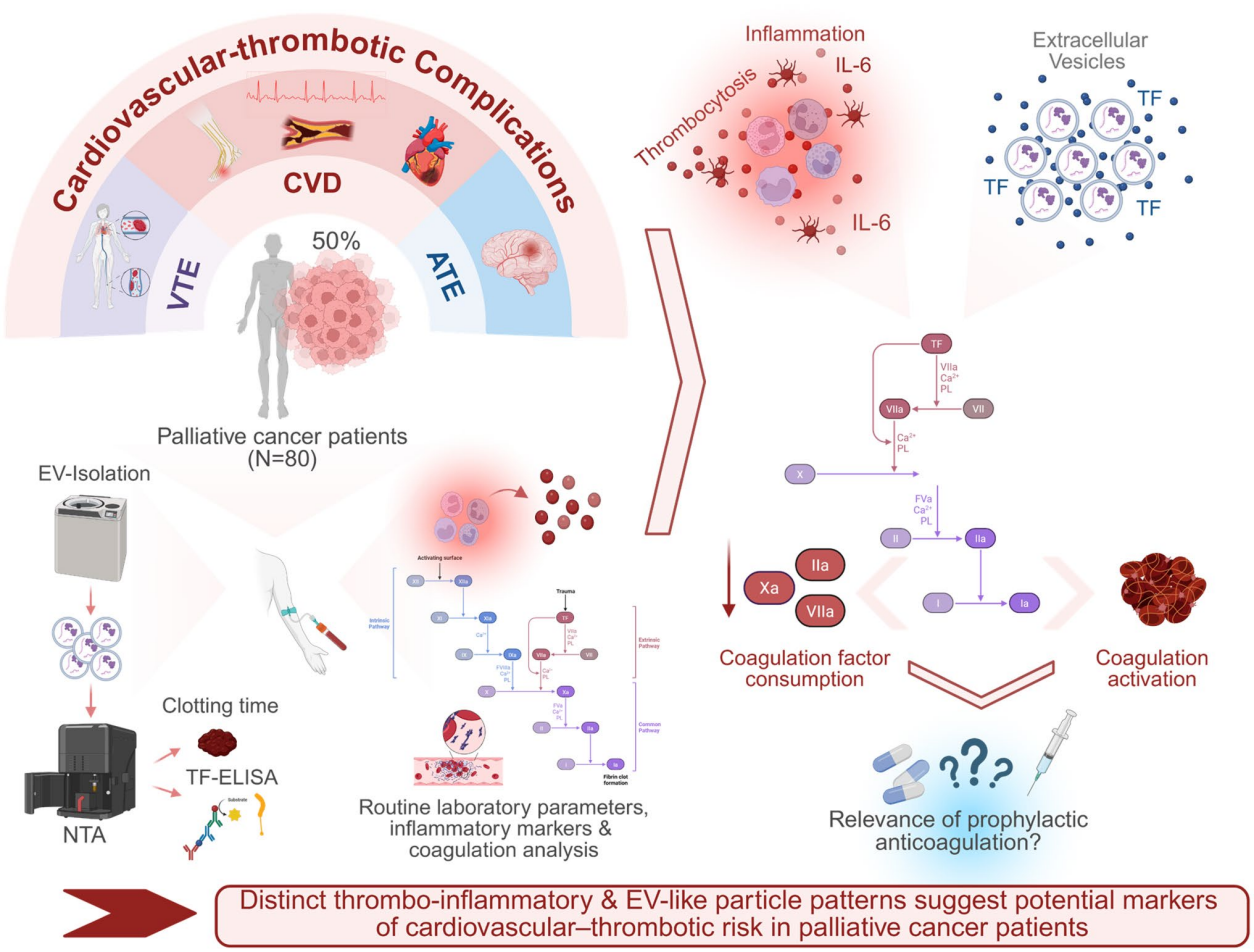

Coagulation-inflammation axis in palliative cancer patients. The graphic illustrates the link between inflammation (↑ IL-6/CRP) and coagulation changes (prolonged clotting, factor depletion, ↑ platelets) in palliative cancer patients. Circulating procoagulant extracellular vesicles (EVs) were detected and highlight the need to reassess anticoagulation efficacy and safety in this high-risk population. (Created in BioRender.)

**Supplementary Information:**

The online version contains supplementary material available at 10.1007/s11239-026-03241-3.

## Highlights

Inflammation and coagulation markers vary widely in palliative cancer patients.

CVTCs associate with coagulation factor depletion and elevated IL-6 levels.

Lower FII activity, higher age, and platelet levels associate with CVTCs.

Effects of anticoagulation in this setting remain unclear.

Cancer patients show increased small EV–like particle concentrations.

**Supplementary Information:**

The online version contains supplementary material available at 10.1007/s11239-026-03241-3.

## Introduction

Cardiovascular-thrombotic complications (CVTCs) are common in cancer patients, particularly in advanced disease stages. CVTCs include venous thromboembolism (VTE), arterial thromboembolism (ATE), and cardiovascular diseases (CVD). CVTCs represent the second leading cause of mortality after cancer itself and significantly impair prognosis by limiting essential oncological treatments [1, 2].

The risk of CVTC in cancer patients is 2- to 7-fold higher than in the general population, affecting up to 30 % of individuals, and occurs even more frequently in patients with advanced-stage disease [3–5]. Although the underlying mechanisms of venous, arterial, and cardiovascular events differ, several biological pathways overlap, and many contributing factors are shared. Alterations in hematologic parameters, such as leukocytosis and thrombocytosis—potentially induced by proinflammatory cytokines like interleukin-6 (IL-6)—have been associated with an increased risk of CVTCs [6–10]. Uncontrolled tumor growth is frequently associated with systemic inflammation and hypercoagulation, further promoting CVTC development.

Procoagulant extracellular vesicles (EVs) — lipid bilayer-enclosed particles released by various cell types — have emerged as promising biomarkers for cancer-associated CVTC. Their potential clinical relevance is particularly noteworthy given the limited specificity of established biomarkers, such as D-dimers [11]. However, despite the high burden of CVTCs in patients with advanced malignancies, few studies have systematically examined their prevalence and associated biological alterations in the palliative care setting [12]. Furthermore, clear guidelines for prophylactic or therapeutic anticoagulant therapy in this patient population are lacking [11, 13]. As many patients receiving palliative therapy survive for extended periods, an improved understanding of coagulation status may support more individualized risk assessment.

In advanced cancer, accumulating evidence points toward sustained hyperactivation of the coagulation–inflammation axis, characterized by elevated inflammatory cytokines, altered coagulation factor activity, and increased release of EVs [14–17]. Yet, the interplay between these components remains insufficiently understood, particularly in palliative-stage disease, where clinical status, therapeutic exposure, and disease dynamics differ substantially from earlier treatment lines. Importantly, very limited data integrate inflammatory markers, coagulation parameters, and EV-associated characteristics within this vulnerable population.

To address this gap, the present study provides an exploratory, retrospective cross-sectional analysis of coagulation parameters, inflammatory markers, and small EV-like particles in palliative cancer patients with and without a history of CVTCs. Given the single-timepoint design, the study aims to identify associative patterns rather than mechanistic pathways. As many patients receive therapeutic anticoagulation, potential influences of antithrombotic therapy were considered descriptively. The findings are intended to generate hypotheses that can guide future longitudinal or interventional research.

## Methods

### Patient recruitment and study design

**Fig. 1:**
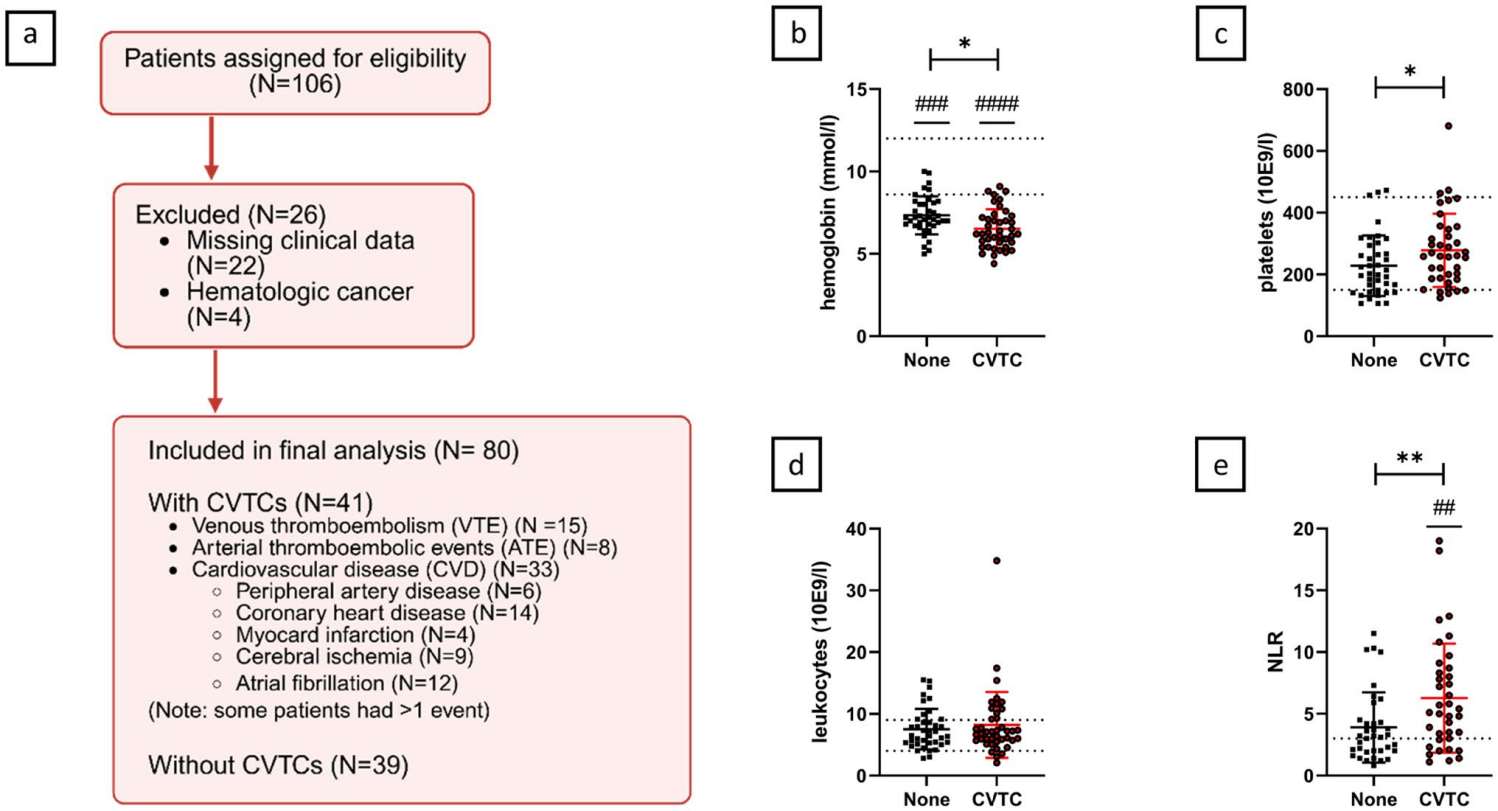
Comparison of hematologic parameters between palliative tumor patients with and without CVTC (scatter dot plots, mean ± SD) (**a**) Patient inclusion and exclusion (**b**) Hemoglobin levels (mmol/l) (**c**) Platelet counts (10⁹/l) (**d**) Leukocyte counts (10⁹/l) (**e**) Neutrophil-to-lymphocyte ratio (NLR). Statistical tests: one-way ANOVA or Kruskal–Wallis test; *p < 0.05, **p < 0.01, ###p < 0.001, ####p < 0.0001 vs. indicated groups. The area between or below the dashed lines represents the standard reference range

This retrospective cross-sectional study included adult participants (> 18 years) with a confirmed diagnosis of solid malignancy in a palliative setting. The local ethics committee reviewed and approved the study (Rostock University Medical Centre, A 2024-0068), and all procedures adhered to the principles outlined in the Declaration of Helsinki. Inclusion was based on written informed consent and additional verbal counseling, irrespective of prior therapies or pre-existing conditions. To reduce selection bias, patients were recruited from both the inpatient and outpatient departments of the clinic of Hematology, Oncology, and Palliative Medicine at Rostock University Medical Centre, resulting in expected clinical heterogeneity in disease status and therapeutic exposures. No formal a priori sample size calculation was conducted. Given the exploratory design, the sample size was determined by patient availability during the study period. A post-hoc estimation based on the observed effect size for FII activity (Cohen’s d = 1.30) suggests sufficient power to detect large group differences. All eligible patients treated in the department between June 2024 and December 2024 were included. Addressing information bias, relevant clinical data were collected from patients’ files, as well as from structured medical history sheets, including age, sex category, tumor entity, metastatic status, date of initial diagnosis, prior oncological treatments, CVD, including ATE or VTE, cardiovascular risk factors, smoking status, body mass index, and anticoagulant therapy (dose and type). The timing of the last anticoagulant administration was not systematically documented and therefore could not be analyzed. A total of 106 palliative cancer patients were screened, of whom 80 met the inclusion criteria and were included in the final analysis. Patients with incomplete clinical data were excluded. The inclusion and exclusion process is illustrated in Fig 1. CVTCs were defined as the presence of at least one of the following: CVD (including coronary heart disease (CHD), myocardial infarction, peripheral arterial disease (PAD), atrial fibrillation), ATE (ischemic stroke), or VTE (including deep vein thrombosis and pulmonary embolism). Due to the heterogeneity and limited number of individual events, these outcomes were analyzed descriptively and additionally combined into a composite endpoint. Patients could present with more than one CVD diagnosis; however, such cases were counted only once within the CVD category. Likewise, individuals with both CVD and VTE were assigned to the CVTC group but counted only once for prevalence analyses. We compared palliative patients with CVTCs to those without these complications to determine clinical and paraclinical differences. The timing of CVTC relative to the initiation of palliative care could not be reliably determined from retrospective documentation and was therefore not analyzed.

Blood samples were collected once at a single, clinically unanchored time point as part of routine laboratory diagnostics and subsequently analyzed in the clinical chemistry laboratory at Rostock University Medical Centre, allowing a cross-sectional assessment of coagulation and inflammatory parameters. Sampling was not standardized with respect to the timing of the thrombotic event, disease progression, inflammatory status, or administration of anticoagulant medication. Documentation of the time interval since the last anticoagulant dose was incomplete and thus interpreted with caution. Potential pharmacodynamic influences of anticoagulant therapy were considered in sensitivity analyses. Additionally, citrate-anticoagulated blood was centrifuged (2,000 × *g*, 10 min) to remove blood cells, and the resulting platelet-poor plasma (PPP) was collected and stored at -80 °C for subsequent EV isolation.

### Laboratory and coagulation analyses

Routine laboratory parameters, including differential blood count, transaminases, cholestasis markers, albumin, and inflammatory markers such as C-reactive protein (CRP) and IL-6, were analyzed from the collected blood samples. Coagulation parameters, including activated partial thromboplastin time (aPTT), prothrombin time (PT) expressed as the International Normalized Ratio (INR), and thrombin clotting time, were also assessed. Additionally, d-dimer levels, fibrinogen concentration, and antithrombin III (AT III) activity were measured. Furthermore, individual coagulation factor activity was determined using coagulometry, referenced against standardized controls. As detailed above, the timing of anticoagulant medication relative to blood sampling was not documented, which limits the interpretation of potential pharmacodynamic effects on coagulation parameters.

### Extracellular vesicle (EVs) isolation and nanoparticle tracking analysis (NTA)

EV-enriched fractions were isolated from 1 ml of stored PPP using differential centrifugation. PPP samples were thawed at room temperature and handled gently to avoid vesicle disruption. PPP was diluted 1:2 with phosphate-buffered saline (PBS) and subjected to a medium-speed centrifugation at 16,000 × g for 10 min at 4 °C to remove cell debris and larger particles. The resulting supernatant, containing EVs, was carefully transferred to an ultracentrifugation tube and topped up with PBS. EV isolation was performed by high-speed ultracentrifugation using a Beckman Coulter Optima XPN-80 ultracentrifuge equipped with an SW40 Ti swinging-bucket rotor at 120,000 × g for 120 min at 4 °C. Following centrifugation, the supernatant was removed, leaving an EV pellet, which was resuspended in PBS and stored at −80 °C until further analysis. EVs were quantified by nanoparticle tracking analysis (NTA) to determine EV concentration and size distribution using a NanoSight® LM10 (Malvern Panalytical, Malvern, United Kingdom). EVs were diluted in PBS and injected into the measurement chamber. A thermometer was used to monitor potential temperature fluctuations. Video recordings were acquired in five consecutive 30-second runs, and mean values for EV concentration and predominant particle size were calculated using NTA 3.3 software. To account for the background signal, PBS particles were measured separately and subtracted from the sample data. Although the applied 16,000 × g step effectively removes most residual platelets and cellular contaminants, plasma samples underwent only a single low-speed centrifugation before freezing. Therefore, minimal platelet-derived contamination cannot be completely excluded, which may contribute to particle counts detected by NTA after thawing. Furthermore, the 16,000 × g step may have partially depleted larger vesicle subpopulations (>400 nm), potentially influencing overall EV quantification and size distribution obtained by NTA, whose upper detection limit lies in a similar range. All isolation steps were performed according to MISEV2023 recommendations, with attention to minimizing pre-analytical variability. As this study focused on a retrospective cross-sectional assessment, EV characterization was limited to NTA and functional assays. No protein-based EV identity markers or morphological analyses were performed, and thus, the isolated particles are referred to as small EV-like particles consistent with the MISEV2023 size range rather than definitively confirmed EVs [18]. 

For readability, these particles are referred to as ‘EVs’ throughout the manuscript. For comparison, PPP was collected from a small group of age-matched healthy donors (n = 12) using the same processing protocol. EV-like particles were isolated using identical procedures to ensure methodological consistency between patient and control samples.

### Clotting of EVs with normal plasma and measurement of tissue factor (TF) abundance on EVs

The clotting time of normal plasma and EVs was quantified using a coagulometer. EVs were diluted in PBS to a final concentration of 1 × 10⁹ particles/ml. For clotting measurements, normal plasma, the EV suspension, and 25 mM CaCl₂ were sequentially added in a coagulometer, and clotting time was recorded. TF abundance on EVs was quantified using a sandwich ELISA (Elabscience®, Catalog No: E-EL-H0040) with isolated patient-derived EVs, following the manufacturer’s instructions. Optical density was measured spectrophotometrically at a wavelength of 450 nm using a Tecan microplate reader. As the assay was performed on bulk EV preparations rather than on individual vesicles, results reflect the overall TF content within the EV isolate and not the proportion of TF-positive EVs. Therefore, TF positivity is reported as bulk-associated TF levels, not as single-vesicle expression. Given the cross-sectional design, these functional readouts provide exploratory insights into EV-associated procoagulant activity.

### Statistical evaluation

All values are given as mean ± SD. Quantitative variables such as laboratory and coagulation parameters were analyzed as continuous variables. After proving the assumption of normality (Shapiro–Wilk test), one-way ANOVA (Dunnett’s multiple comparisons), two-way ANOVA (Tukey’s multiple comparisons), or t-test was performed, whereas non-normally distributed variables were analyzed using the Mann–Whitney U-test or Kruskal–Wallis test. Categorical variables were compared using Fisher’s exact test. GraphPad Prism version 8.0.2 (GraphPad Software, San Diego, CA, USA) was used for descriptive and univariate analyses.

The magnitude of the difference in FII activity between patients with and without CVTCs was quantified using Cohen’s d, calculated from group means and the pooled SD.

To explore factors associated with FII activity, a multiple linear regression model was performed with FII as the dependent variable and CVTC status and albumin as independent variables. Model fit was assessed using adjusted R^2^, and multicollinearity was evaluated with variance inflation factors (VIF). A sensitivity analysis excluding patients receiving therapeutic anticoagulation was conducted.

To explore factors associated with the presence of CVTCs, a binary logistic regression analysis was conducted with CVTC status (yes/no) as the dependent variable and FII activity, platelet count, and age as independent variables. Model adequacy was assessed using the Hosmer–Lemeshow test, and variance explanation was summarized with Nagelkerke’s R^2^. A sensitivity model excluding patients on therapeutic anticoagulation was also calculated. Logistic regression analyses were performed using SPSS (IBM SPSS Statistics, IBM Corp., Armonk, NY, USA).

All analyses were exploratory, and due to the cross-sectional design, associations are interpreted as non-causal. A two-sided p-value < 0.05 was considered statistically significant.

## Results

### Patient characteristics and general clinical data

In this study, 80 patients with advanced solid tumors receiving palliative therapy were recruited. Key demographic and clinical characteristics are summarized in Table 1. The mean age was 69 years, with 39 % female participants. Approximately half of the patients were recruited from inpatient care. The most common cancer type was gastrointestinal malignancies, primarily treated with chemotherapy and/or immunotherapy (Table 1). At enrollment, 69 % of patients had distant metastases, most commonly hepatic metastases. Only 12 % of the cohort received palliative best supportive care. Cardiovascular risk (CVR) factors were observed in more than two-thirds of the study population, most commonly arterial hypertension. At the time of inclusion, 57 % of patients received anticoagulant therapy, predominantly LMWH, which was more common in inpatients than outpatients (32.8 % vs. 8.3 %, p = 0.0133).

**Table 1:** Demographic and clinical characteristics

Baseline clinical characteristics	Total (n=80)	Tumor patients without CVTC (n=39)	Tumor patients with CVTC (n=41)	p-value
Age (years)	69.2 ± 11.7	66.2 ± 12.1	72.3 ± 10.3	**0.034**
Sex category (number, %)				
Female	31 (38.8 %)	19 (48.7 %)	12 (29.3 %)	0.107
Male	49 (61.3 %)	20 (51.3%)	29 (70.7 %)	
Recruitment				
Inpatient	44 (55 %)	18 (46.2 %)	26 (63.4 %)	0.177
Outpatient	36 (45 %)	21 (53.8 %)	15 (36.6 %)	
Cancer types (number, %)				
Gastrointestinal-Ca	48 (61.5 %)	24 (61.5 %)	22 (53.7 %)	0.505
Hepatocellular-Ca	6 (7.7 %)	3 (7.7 %)	3 (7.3 %)	
Cholangiocellular -Ca	11 (14.1 %)	3 (7.7 %)	6 (14.6 %)	
Colorectal-Ca	13 (16.7 %)	8 (20.5 %)	5 (12.2 %)	
Pancreatic-Ca	14 (17.9 %)	8 (20.5 %)	6 (14.6 %)	
Gastric-Ca	4 (5.1 %)	2 (5.1 %)	2 (4.9 %)	
Urogenital-Ca	9 (11.5 %)	3 (7.7 %)	6 (14.6 %)	0.483
Prostate-Ca	2 (2.6 %)	1 (2.6%)	1 (2.4 %)	
Urothelial-Ca	2 (2.6 %)	0	2 (4.9 %)	
Renal-Ca	5 (6.4 %)	2 (5.1 %)	3 (7.3 %)	
Bronchial-Ca	7 (9 %)	5 (12.8 %)	2 (4.9 %)	0.259
Breast-Ca	3 (3.8 %)	2 (5.1 %)	1 (2.4 %)	1.000
Others	11 (14.1 %)	2 (5.1 %)	6 (14.6 %)	0.265
Malignant Melanoma	1 (1.3 %)	1 (2.6 %)	0	
Oropharyngeal-Ca	2 (2.6 %)	0	2 (4.9 %)	
Sarkoma	2 (2.6 %)	1 (2.6 %)	1 (2.4 %)	
Thyreoid-Ca	1 (1.3 %)	1 (2.6 %)	0	
Cancer of unkown origin	5 (6.4 %)	2 (5.1 %)	3 (7.3 %)	
Metastasis (number, %)	55 (68.8 %)	26 (66.7 %)	29 (70.7 %)	0.810
Hepatic	35 (43.8 %)	19 (48.7 %)	15 (36.6 %)	
Pulmonal	19 (23.8 %)	16 (41 %)	3 (7.3 %)	
Cerebral	12 (15 %)	6 (15.4 %)	6 (14.6 %)	
Ossary	11 (13.8 %)	6 (15.4 %)	5 (12.2 %)	
Cardial	1 (1.3 %)	0	1 (2.4 %)	
Oncological treatment (number, %)				–
Surgery	30 (37.5 %)	17 (43.6 %)	14 (34.1 %)	0.491
Radiation	10 (12.5 %)	5 (12.8 %)	5 (12.2 %)	1.000
Chemotherapy	50 (62.5 %)	27 (69.2 %)	24 (58.5 %)	0.359
Immunotherapy	41 (51.3 %)	21 (53.8 %)	20 (48.8 %)	0.662
Best supportive care	10 (12.5 %)	1 (2.6 %)	6 (14.6 %)	0.109
Venous thromboembolic events (number, %)	15 (18.8 %)	–	–	–
Deep vein thrombosis	9 (11.3 %)			
Pulmonary artery embolism	4 (5 %)			
Others (Portal vein thrombosis, Sinus vein thrombosis)	2 (2.3 %)			
Cardiovascular Disease (number, %)	25 (30 %)	–	–	–
Peripheral artery disease	6 (7.5 %)			
Myocardial infarction	4 (5 %)			
Coronary heart disease	14 (17.5 %)			
Atrial fibrillation	12 (15 %)			
Arterial thromboembolism (number, %)	9 (11.3 %)			
Cerebral ischaemia	9 (11.3 %)			
Cardiovascular risk factors	53 (66.3 %)	19 (48.7 %)	34 (82.9 %)	**0.002**
Arterial Hypertension	47 (58.8 %)	15 (38.5 %)	32 (78 %)	**0.001**
Diabetes mellitus	20 (25 %)	10 (25.6 %)	9 (22 %)	0.795
BMI > 30 kg/m^2^	16 (20 %)	7 (17.9 %)	9 (22 %)	0.782
Smoking	44 (55 %)	19 (48.7 %)	22 (53.7 %)	0.823
Anticoagulative treatment	45 (56.3 %)	14 (35.9 %)	31 (75.6 %)	**0.001**
Prophylactic low molecular weight heparin	17 (21.3 %)	11 (28.2 %)	6 (14.6 %)	0.276
Therapeutic low molecular weight heparin	12 (15 %)	0	11 (26.8 %)	**0.001**
Direct oral anticoagulants (DOAC)	7 (8.8 %)	0	7 (17.1 %)	**0.012**
Antiplatelet drug	11 (13.8 %)	4 (10.3 %)	7 (17.1 %)	0.519

Demographic and clinical characteristics of the palliative cancer cohort (n = 80), comparing patients with CVTC (n = 41) and without CVTC (n = 39). Data are presented as mean ± SD or frequency (%). Statistical differences between groups are shown in the last column (p-value). Anticoagulation status reflects therapy at the time of blood sampling; detailed timing and dosing information were not available

Bold values indicate statistical significance (p < 0.05)

Overall, 41 of 80 patients (51 %) had a documented history of CVTCs. VTE was present in 19 %, CVD in 30 %, and ATE in 11 % of patients; overlapping diagnoses resulted in 41 unique individuals in the CVTC group. Patients with CVTC were significantly older than those without CVTC, while no significant group differences were observed in terms of sex distribution, cancer type, or metastatic status. Cardiovascular risk factors, particularly arterial hypertension, were more prevalent in the CVTC group. Anticoagulant therapy was also more frequent in patients with CVTC: 76 % received anticoagulation, including 15 % prophylactic LMWH. Among patients without CVTC, 28.2 % received prophylactic anticoagulation. Therapeutic anticoagulation with LMWH or DOACs was administered only to patients with previous CVTCs (Table 1).

### Hematologic patterns in palliative cancer patients with cardiovascular-thrombotic complications (CVTC)

To investigate the differences in hematological, proinflammatory, and coagulation markers between cancer patients with and without CVTCs, various laboratory parameters were analyzed. Notably, hemoglobin levels were significantly lower in patients with CVTC, despite being reduced in nearly all patients (Fig. 1b). Platelet counts, while mostly within the normal range, were significantly higher in cancer patients with CVTC (Fig. 1c). No significant difference was observed in leukocyte counts (Fig. 1d); however, the neutrophil-to-lymphocyte ratio (NLR) was elevated in patients with a history of CVTC (Fig. 1e).

**Fig. 2:**
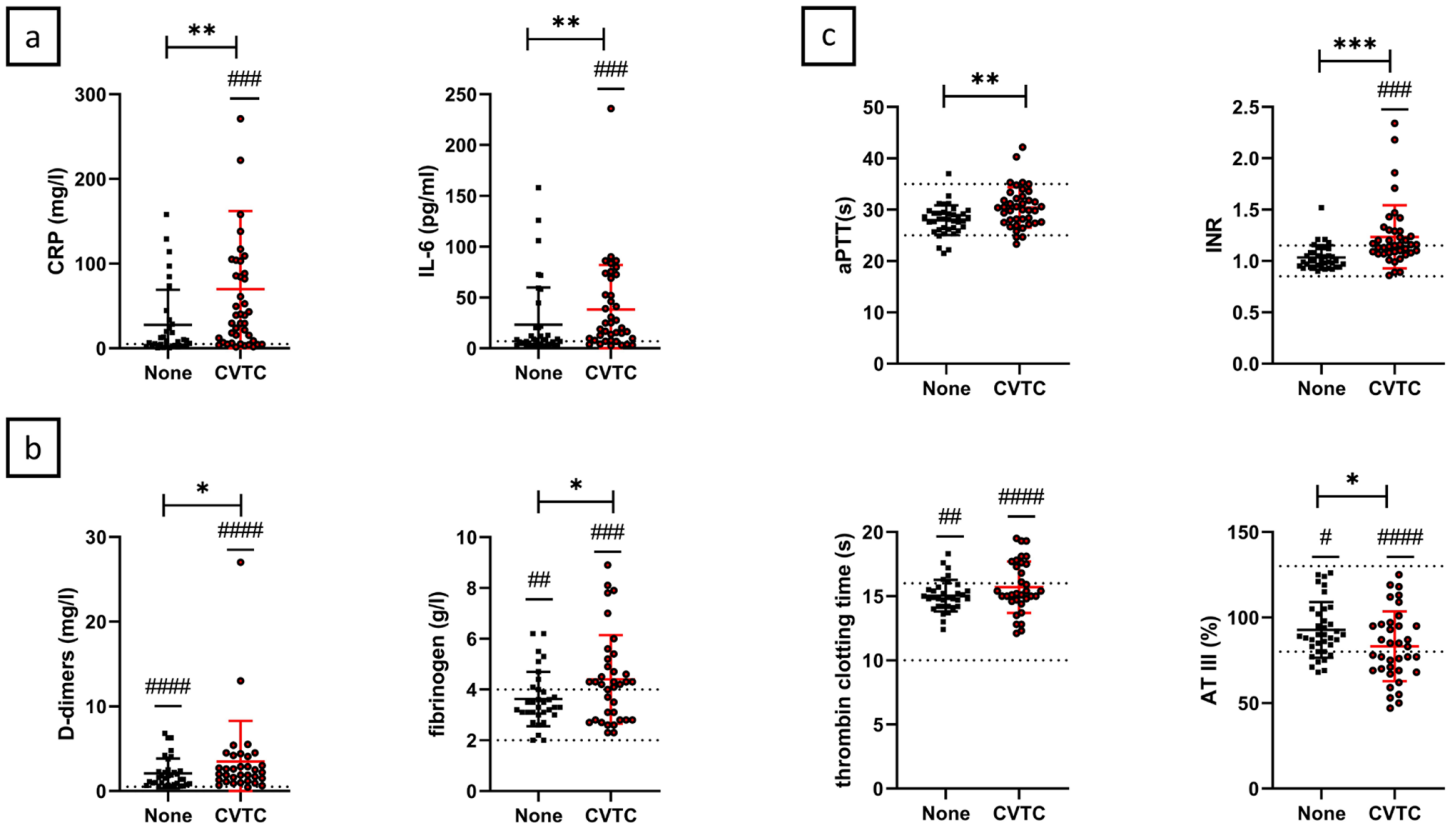
Proinflammatory markers and clotting times (scatter dot plots, mean ± SD) (**a**) CRP (mg/l) and IL-6 (pg/ml) (**b**) D-dimers (mg/l) and fibrinogen (g/l) (**c**) aPTT, INR, thrombin clotting time, and AT III activity. Statistical tests: one-way ANOVA, Mann–Whitney test, or Kruskal–Wallis test; *p < 0.05, **p < 0.01, ***p < 0.001, #p < 0.05, ##p < 0.01, ###p < 0.001, ####p < 0.0001 vs. indicated groups or reference range The area below or between the dashed lines represents the standard reference range

Proinflammatory markers, including CRP and IL-6, were significantly higher in patients with CVTC (Fig. 2a). Fibrinogen and D-dimer levels were elevated in the overall cohort and showed significantly higher values in patients with CVTC compared to those without CVTC (Fig. 2b). Regarding global coagulation parameters, aPTT and PT/INR were significantly prolonged in patients with CVTC (Fig. 2c). Thrombin clotting time was mildly prolonged in both groups without notable differences between them (Fig. 2c).

**Fig. 3:**
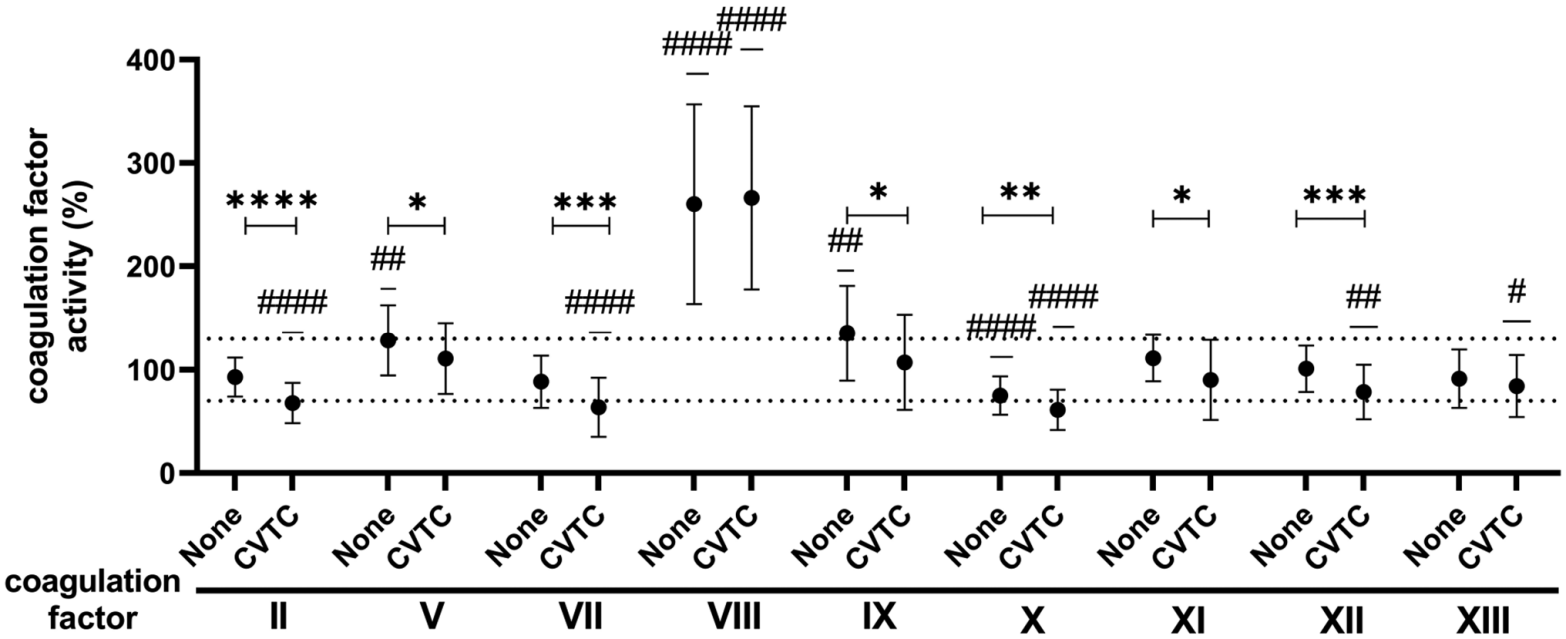
Coagulation factor activity (%) in palliative tumor patients with and without CVTC (mean ± SD) Activities of FII, V, VII, IX, X, XI, XII, and XIII compared between groups and with the reference range. Statistical test: one-way ANOVA; *p < 0.05, **p < 0.01, ***p < 0.001, ****p < 0.0001, #p < 0.05, ##p < 0.01, ####p < 0.0001 vs. indicated groups or reference range The area between the dashed lines represents the standard reference range

Activities of individual coagulation factors and AT III were measured to characterize coagulation profiles in patients with and without CVTC. AT III activity was significantly lower in patients with CVTC (Fig. 2c). Several coagulation factors, including FII, V, VII, IX, X, XI, and XII, showed significantly reduced activity in patients with CVTC compared to those without CVTC (Fig. 3). In the overall cohort, activities of FII, VII, X, XII, and XIII were frequently below the reference range, whereas FVIII activity was markedly elevated in most patients (Fig. 3).

**Fig. 4:**
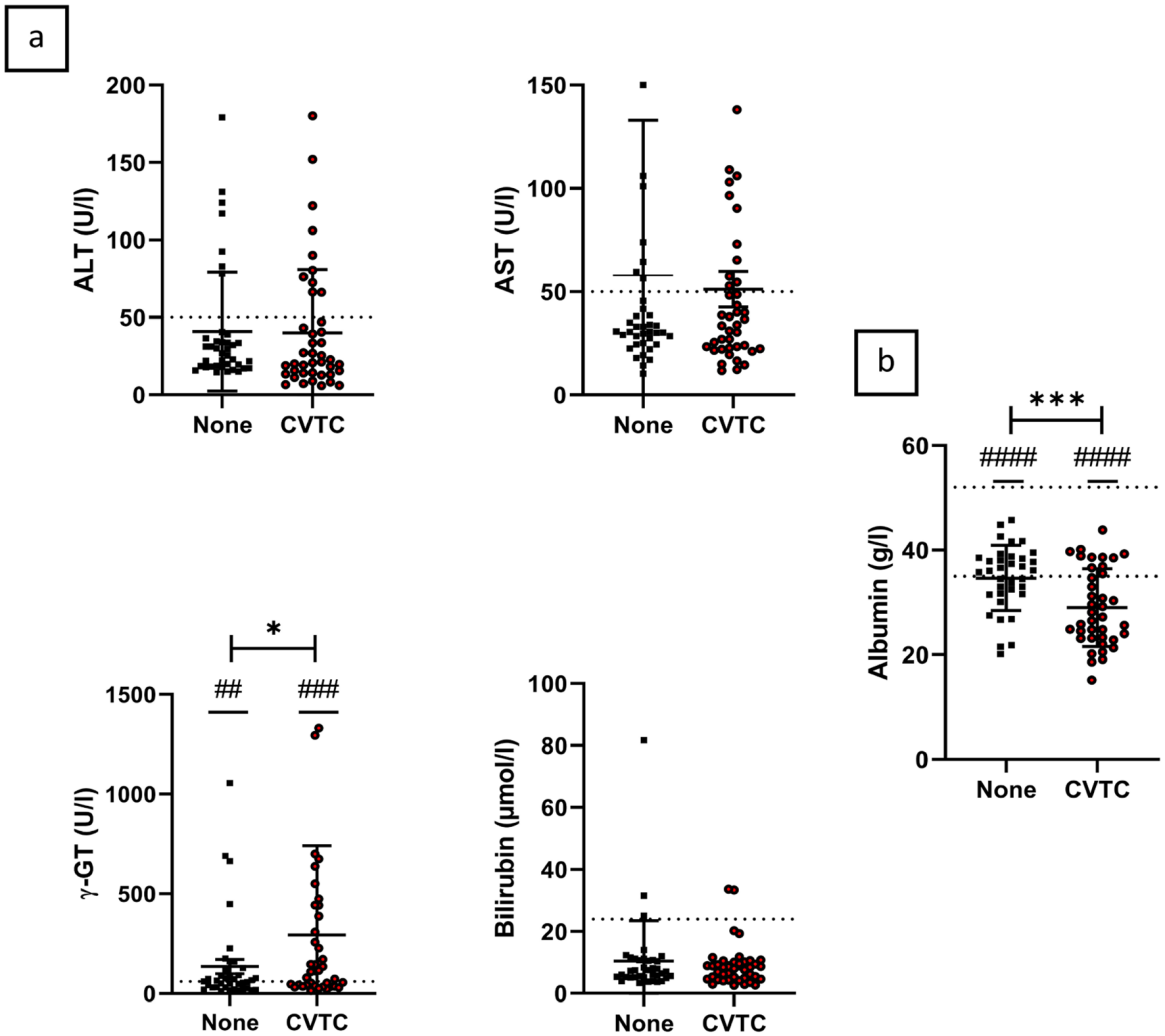
Liver function parameters in patients with and without CVTC (scatter dot plots, mean ± SD**)** (**a**) Transaminase activity (U/l), bilirubin (µmol/l), and γ-GT activity (U/l) (**b**) Albumin concentration (g/l). Statistical tests: Kruskal–Wallis test and one-way ANOVA; *p < 0.05, **p < 0.01, ***p < 0.001, ###p < 0.001, ####p < 0.0001 vs. indicated groups or reference range The area between the dashed lines represents the standard reference range

Liver function parameters, including transaminases, cholestasis markers, and albumin, were analyzed to characterize hepatic-related laboratory findings in patients with and without CVTC (Fig. 4). No significant difference in the prevalence of hepatic metastases was observed between the two groups (Table 1). γ-GT levels were higher in patients with CVTC compared to those without CVTC. Albumin levels were reduced in the overall cohort and were significantly lower in patients with CVTC (Fig. 4).

To characterize factors associated with coagulation factor activity, an exploratory multiple linear regression was performed with FII activity as the dependent variable (Sup.Table 1). In the main model, the presence of CVTC was associated with lower FII activity (B = –25.9, 95% CI –40.72 to –11.11; p < 0.001). Albumin concentration and anticoagulant therapy showed no significant associations with FII activity. In a sensitivity analysis excluding patients receiving therapeutic anticoagulation, the association between CVTC and lower FII activity remained (Sup. Table 1).

A binary logistic regression analysis was conducted to explore associations between selected clinical and laboratory parameters and the presence of CVTCs (Supplementary Table 2). In the final model, lower FII activity (OR 0.91, 95% CI 0.87–0.96; p < 0.001), higher age (OR 1.10, 95% CI 1.01–1.20; p = 0.031), and higher platelet count (OR 1.01, 95% CI 1.00–1.02; p = 0.091) were associated with CVTCs. A sensitivity analysis excluding patients receiving therapeutic anticoagulation yielded comparable results, with FII activity remaining significantly associated with CVTCs. Full regression coefficients, confidence intervals, and model diagnostics are reported in Sup. Table 2.

### Procoagulant extracellular vesicles (EVs) are elevated in palliative cancer patients

**Fig. 5:**
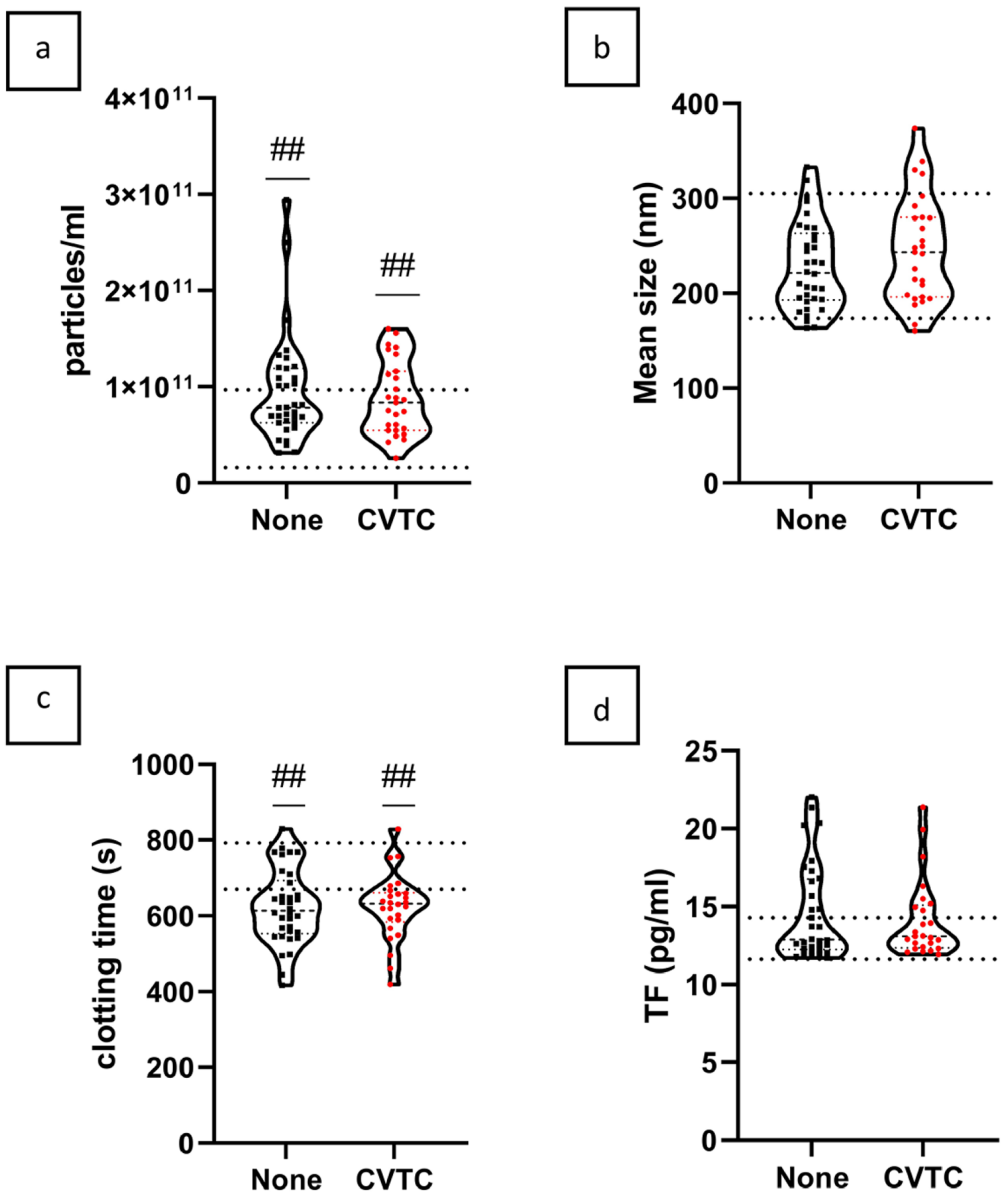
Extracellular vesicle (EV) concentration, mean size, and procoagulant activity (violin plots, mean ± SD) (**a**) EV concentration (particles/ml) (**b**) Mean size (nm) (**c**) Clotting time (s) (**d**) Tissue factor (TF) abundance (pg/ml). Statistical test: Kruskal–Wallis test; ##p < 0.01 vs. reference range The area between the dashed lines represents the standard reference range of healthy donors

EVs were analyzed to compare EV-related parameters between patients with and without CVTCs. EVs from 20 age- and sex-matched healthy donors served as controls. Cancer patients showed significantly higher EV concentrations compared to healthy donors (Fig. 5). However, no significant difference in EV concentration was observed between patients with and without CVTCs. Mean EV size was similar across all groups and ranged from 150 to 400 nm (Fig. 5).

Functional assessment of EVs showed that EV-associated clotting time was significantly shorter in cancer patients than in healthy donors, while no significant differences were observed between cancer patients with and without CVTCs (Fig. 5). TF abundance in EV preparations was also measured. EV-associated TF levels were slightly higher in cancer patients than in healthy donors, but this difference was not statistically significant (Fig. 5).

## Discussion

In this exploratory cross-sectional study of 80 patients with advanced solid tumors receiving palliative care, approximately half had a history of CVTCs. Patients with CVTCs showed higher levels of inflammatory markers and differences in hematologic and coagulation parameters, including prolonged clotting times and lower activities of several coagulation factors. Fibrinogen and d-dimer levels were elevated in the overall cohort and were higher in patients with CVTCs. In regression analyses, lower FII activity and higher platelet counts were associated with CVTCs. Cancer patients also exhibited higher concentrations of small EV–like particles than healthy donors, although no EV-related differences were observed between patients with and without CVTCs.

The cohort predominantly consisted of older patients with gastrointestinal tumors who had received various systemic treatments, including chemotherapy and immunotherapy. CVD was more frequent than VTE, and almost half of the patients presented with at least one cardiovascular comorbidity. This prevalence is higher than previously reported in population-based studies, although direct comparisons are limited by differences in study design and patient populations, particularly the inclusion of individuals receiving palliative care [19]. CVD increases morbidity, mortality, and treatment discontinuations, yet its significance in advanced cancer remains understudied. Research on its management and pathogenesis is also lacking compared to VTE [19–21]. Patients with CVD, ATE and/or VTE were combined into a single CVTC group to reflect the clinical overlap of thrombotic manifestations in advanced cancer; however, this approach may introduce heterogeneity, including differences in the type and indication for antithrombotic therapies across these subgroups. No significant differences in tumor entities, metastatic status, or prior oncological treatments were observed between patients with and without CVTCs. Although certain cancer types and anticancer therapies have been associated with thrombotic risk in other studies, such associations were not apparent in our cohort [22–24]. This may reflect the advanced disease stage and diverse treatment trajectories of palliative patients, which can obscure tumor- or therapy-specific risk patterns.

Marked heterogeneity in coagulation parameters was observed across the cohort, with patients with CVTCs showing more pronounced deviations. These included prolonged coagulation times and lower activities of several coagulation factors. AT III activity was also reduced in patients with CVTCs. Thrombin clotting time was mildly prolonged in both groups, consistent with the advanced disease stage of the cohort. Exploratory regression analyses were performed to investigate factors associated with FII activity and the presence of CVTCs. In the linear regression model, CVTC status was associated with lower FII activity, whereas albumin levels and anticoagulant use showed no significant associations; similar results were obtained in a sensitivity analysis excluding patients on therapeutic anticoagulation. These results indicate that the lower FII activity observed in patients with CVTCs cannot be readily attributed to anticoagulant use or hepatic synthetic function within the limitations of this study design. In the logistic regression model, lower FII activity and higher age were associated with CVTCs, while platelet count demonstrated a statistical trend. These findings are consistent with previous reports linking reduced coagulation factor activity and higher platelet levels to thrombotic risk in malignancy, although the directionality and underlying contributors cannot be determined in this study [10]. The wide confidence intervals for some variables underscore the need for cautious interpretation and reflect the limited sample size and heterogeneity of the cohort. Given that more than half of the cohort and the majority of patients with CVTCs received anticoagulant therapy, the interaction between anticoagulation and the heterogeneous coagulation profiles observed in advanced malignancy remains insufficiently understood [25–27]. Anticoagulant use was not significantly associated with FII activity, but the cross-sectional design does not allow conclusions about its influence on coagulation factors.

Although several coagulation factors were reduced, the clinical relevance and underlying contributors of these findings cannot be determined within this cross-sectional study. Lower factor levels could be relevant for bleeding, particularly in patients receiving anticoagulation, but bleeding outcomes were not assessed, and no causal inferences can be made. Previous studies have reported increased bleeding rates in palliative cancer patients receiving anticoagulation, although the contribution of factor depletion to this risk remains unclear [12, 28]. Future longitudinal studies integrating serial coagulation measurements with standardized documentation of bleeding events will be needed to clarify whether reduced factor activities meaningfully influence bleeding susceptibility in this setting and to evaluate how coagulation status may interact with the effectiveness and safety of different anticoagulant regimens [29].

Markers of fibrinolysis and inflammation were elevated throughout the cohort, consistent with the advanced disease stage of the included patients. Fibrinogen and d-dimer levels were increased in nearly all individuals and were higher in patients with CVTCs. Elevated d-dimer concentrations have previously been associated with poorer prognosis in patients with advanced cancer. However, the underlying determinants of these elevations are multifactorial and cannot be clarified within the present study design [30, 31].

Proinflammatory markers, including CRP and IL-6, were also higher in patients with CVTCs. Elevated IL-6 levels have been associated with reduced overall survival in several cancer populations [32] and have been linked to cardiovascular and thrombotic conditions such as atherosclerosis, coronary artery disease, and atrial fibrillation [17, 33]. However, the temporal and biological relationships between inflammation, coagulation alterations, and CVTCs cannot be established from this cross-sectional analysis. It therefore remains unclear whether inflammatory activity precedes or follows thrombotic events, or whether both reflect the overall disease burden characteristic of advanced malignancy. Additionally, differences in clinical status, treatment exposure, and disease trajectories between inpatients and outpatients may have contributed to the variability observed in inflammatory and coagulation parameters. Taken together, these findings indicate that elevated inflammatory and fibrinolytic markers are frequent in palliative cancer patients and appear more pronounced in those with CVTCs, although the directionality and mechanistic pathways underlying these patterns remain unresolved.

Extracellular vesicle–like particles were detectable in all patient samples and were elevated in cancer patients compared with healthy donors, which aligns with previous observations in malignant disease. [34–37]. However, EV-related parameters did not differ between patients with and without CVTCs. Interpretation of these findings is limited by methodological factors, including possible platelet-derived contamination, the depletion of larger vesicles during centrifugation, and the lack of protein-based EV markers or imaging methods as recommended by MISEV2023 [18]. TF abundance showed no significant group differences, and the absence of standardized TF assays further restricts conclusions [38]. Overall, while EV concentrations appeared elevated in advanced cancer, the clinical relevance of these observations remains uncertain, underscoring the need for standardized and longitudinal EV studies to clarify their potential association with coagulation changes in this population.

This study has several limitations. Its cross-sectional design and single-time-point sampling do not allow conclusions about temporal or causal relationships between inflammation, coagulation parameters, and CVTCs. The cohort was heterogeneous, including inpatient and outpatient populations with variable disease trajectories and treatments, and the timing, dosing, and indication of anticoagulation were not systematically documented. The exploratory regression analyses were limited by the number of events and should be interpreted with caution. EV characterization was restricted to NTA and bulk TF measurement without confirmatory markers, and residual platelet contamination or loss of larger vesicles cannot be excluded. In addition, the healthy donor group was small and not matched to the patient cohort. As a single-center study with a modest sample size, generalizability may be limited.

In this exploratory cross-sectional study of palliative care patients with advanced solid tumors, CVTCs were associated with differences in inflammatory markers, platelet counts, and selected coagulation factor activities. These findings illustrate the complexity and variability of hemostatic alterations in advanced cancer but do not allow conclusions regarding underlying mechanisms or clinical causality. While elevated EV concentrations and changes in coagulation parameters were observed at the group level, their clinical relevance remains uncertain and requires validation in standardized, longitudinal studies. Overall, this work contributes preliminary descriptive data that may inform future hypothesis-generating research aimed at understanding coagulation disturbances in advanced malignancy.

## Supplementary Information

Below is the link to the electronic supplementary material.

Supplementary file1

Supplementary file2

## Data Availability

The data that support the findings of this study are available from the corresponding author upon reasonable request.

## Abbreviations

aPTT: Activated Partial Thromboplastin Time
ATE: Arterial Thromboembolism
AT III: Antithrombin III
CI: Confidence Interval
CHD: Coronary Heart Disease
CRP: C-reactive Protein
CVTC: Cardiovascular-Thrombotic Complications
CVD: Cardiovascular Disease
CVR: Cardiovascular Risk
DIC: Disseminated Intravascular Coagulation
EVs: Extracellular Vesicles
FII: Coagulation factor II
IL-6: Interleukin-6
INR: International Normalized Ratio
LMWH: Low-Molecular-Weight Heparin
NLR: Neutrophil-Lymphocyte-Ratio
NTA: Nanoparticle Tracking Analysis
OR: Odds Ratio
PAD: Peripheral Arterial Disease
PBS: Phosphate-Buffered Saline
PPP: Platelet-Poor Plasma
PT: Prothrombin Time
TF: Tissue Factor
VTE: Venous Thromboembolic Disease
