# Supplementary material for: The coagulation–inflammation axis in advanced cancer: associations with cardiovascular-thrombotic complications
Source: J Thromb Thrombolysis. 2026 Feb 2;59(4):850–63. doi: 10.1007/s11239-026-03241-3 (PMC13264542; doi:10.1007/s11239-026-03241-3)
Supplement: Supplementary file 1 — Supplementary file1 [file 11239_2026_3241_MOESM1_ESM.docx]

**Supplementary Table 1: Linear regression model for FII activity (main model and sensitivity analysis)**

|  |  |  |  |  |  | |  | |  |
| --- | --- | --- | --- | --- | --- | --- | --- | --- | --- |
| predictors | **Main model B (SE)** | **Main model 95 % CI** | **Main model**  **p-value** | **Sensitivity model B (SE)** | | **Sensitivity model 95% CI** | | **Sensitivity model**  **p-value** | |
| Constant | 87.797 (16.251) | [55.2, 120.4] | <0.001 | 89.971 (29.124) | [29.9, 149.9] | | 0.005 | |  |
| CVTC | -24.117 (6.358) | [-36.9, -11.4] | **<0.001** | -26.873 (10.555) | [-48.6, -5.1] | | **0.017** | |  |
| Anticoagulative treatment | -3.839 (5.747) | [-15.4, 7.7] | 0.507 | - | - | | - | |  |
| Albumin | 0.198  (0.433) | [-0.7, 1.1] | 0.650 | 0.158  (0.774) | [-1.4, 1.8] | | 0.840 | |  |

Linear regression models assessing associations between FII activity and selected clinical variables. The main model included CVTC status and albumin concentration as independent variables and showed an adjusted R² of 0.321. A sensitivity analysis excluding patients receiving therapeutic anticoagulation was performed; in this model, the variable “anticoagulation” was automatically removed due to lack of variance, and the adjusted R² was 0.302. Regression coefficients (B) are presented with standard errors and 95% confidence intervals.
