# Supplementary material for: The coagulation–inflammation axis in advanced cancer: associations with cardiovascular-thrombotic complications
Source: J Thromb Thrombolysis. 2026 Feb 2;59(4):850–63. doi: 10.1007/s11239-026-03241-3 (PMC13264542; doi:10.1007/s11239-026-03241-3)
Supplement: Supplementary file 2 — Supplementary file2 [file 11239_2026_3241_MOESM2_ESM.docx]

**Supplementary Table 2: Binary logistic regression models for predictors of CVTC (main and sensitivity analyses)**

|  |  |  |  |  |  |  |  |  |
| --- | --- | --- | --- | --- | --- | --- | --- | --- |
| predictors | **Main model**  **B (SE)** | **Main model Odds Ratio** | **Main model**  **95% CI** | **Main model p-value** | **Sens. model**  **B (SE)** | **Sens. model Odds Ratio** | **Sens. model 95% CI** | **Sens.**  **p-value** |
| Constant | -0.536  (3.074) | 0.585 |  | 0.862 | 0.066 (5.345) | 1.068 |  | 0.990 |
| Age | 0.097  (0.045) | 1.102 | [1.01, 1.20] | **0.031** | 0.121  (0.083) | 1.128 | [0.96, 1.33] | 0.145 |
| Platelet count | 0.006 (0.003) | 1.006 | [1.00, 1.01] | 0.091 | 0.007 (0.010) | 1.007 | [0.99, 1.03] | 0.463 |
| FII activity | -0.093 (0.027) | 0.911 | [0.87, 0.96] | **<0.001** | -0.142  (0.064) | 0.868 | [0.77, 0.99] | **0.028** |

Binary logistic regression models assessing associations between CVTC occurrence and selected clinical variables. The main model included FII activity, age, and platelet count as independent variables and demonstrated adequate model fit (Hosmer–Lemeshow p = 0.910; Nagelkerke R² = 0.522). A sensitivity analysis excluding patients receiving therapeutic anticoagulation was performed, and model fit remained acceptable (Hosmer–Lemeshow p = 0.936; Nagelkerke R² = 0.622). Odds ratios (OR) are presented with 95% confidence intervals and p-values.
